# ERA Registry Figure of the month Patient survival on dialysis and kidney transplantation

**DOI:** 10.1093/ckj/sfaf051

**Published:** 2025-02-12

**Authors:** Vianda S Stel, Alberto Ortiz, Anneke Kramer

**Affiliations:** ERA Registry, Department of Medical Informatics, Amsterdam UMC – Location, University of Amsterdam, Amsterdam, the Netherlands; Amsterdam Public Health Research Institute, Quality of Care, Amsterdam, the Netherlands; Department of Nephrology and Hypertension, IIS-Fundacion Jimenez Diaz UAM, Madrid, Spain; Department of Medicine, Universidad Autonoma de Madrid, Madrid, Spain; ERA Registry, Department of Medical Informatics, Amsterdam UMC – Location, University of Amsterdam, Amsterdam, the Netherlands; Amsterdam Public Health Research Institute, Quality of Care, Amsterdam, the Netherlands

**Figure 1:**
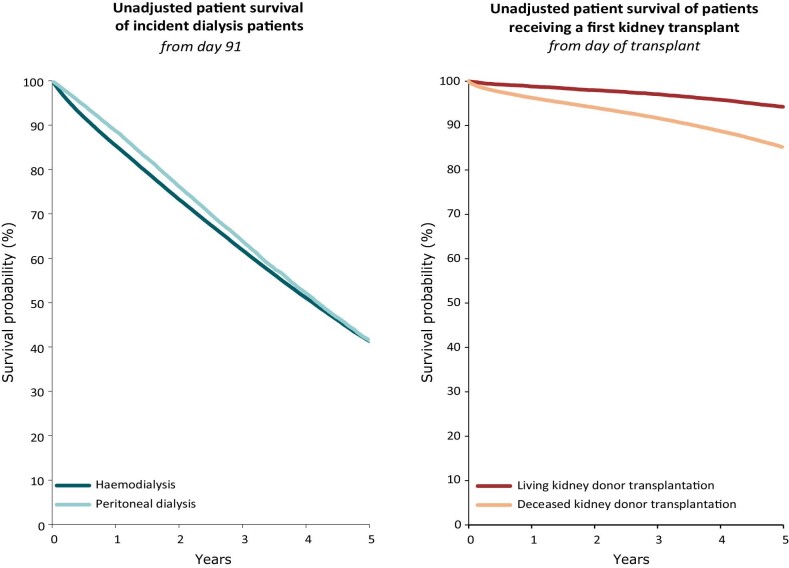
Patient survival for incident dialysis patients by modality (haemodialysis or peritoneal dialysis) from day 91 (cohort 2013–2017) (left panel) and for first-time kidney transplant recipients by donor type (deceased or living) from day of transplantation (cohort 2013–2017) (right panel). **Source:** Boenink et al. CKJ 2024, https://doi.org/10.1093/ckj/sfae405, Figs. 13 and 14. **Explanation:** The unadjusted 5-year survival probability was similar for patients starting haemodialysis (41.3%) and for patients starting peritoneal dialysis (41.5%). In patients receiving a first kidney transplant, the unadjusted 5-year survival was higher for those who received a living donor (94.2%) than for those who received a deceased donor (85.8%).

